# Supplementary material for: The VIP-VPAC2 neuropeptidergic axis is a cellular pacemaking hub of the suprachiasmatic nucleus circadian circuit
Source: Nat Commun. 2020 Jul 7;11:3394. doi: 10.1038/s41467-020-17110-x (PMC7341843; doi:10.1038/s41467-020-17110-x)
Supplement: Supplementary file 1 — Supplementary Information [file 41467_2020_17110_MOESM1_ESM.pdf]

Supplementary Information

The VIP-VPAC2 neuropeptidergic axis is a cellular pacemaking hub of the suprachiasmatic nucleus circadian circuit.

Patton et al.

Supplementary Figure 1

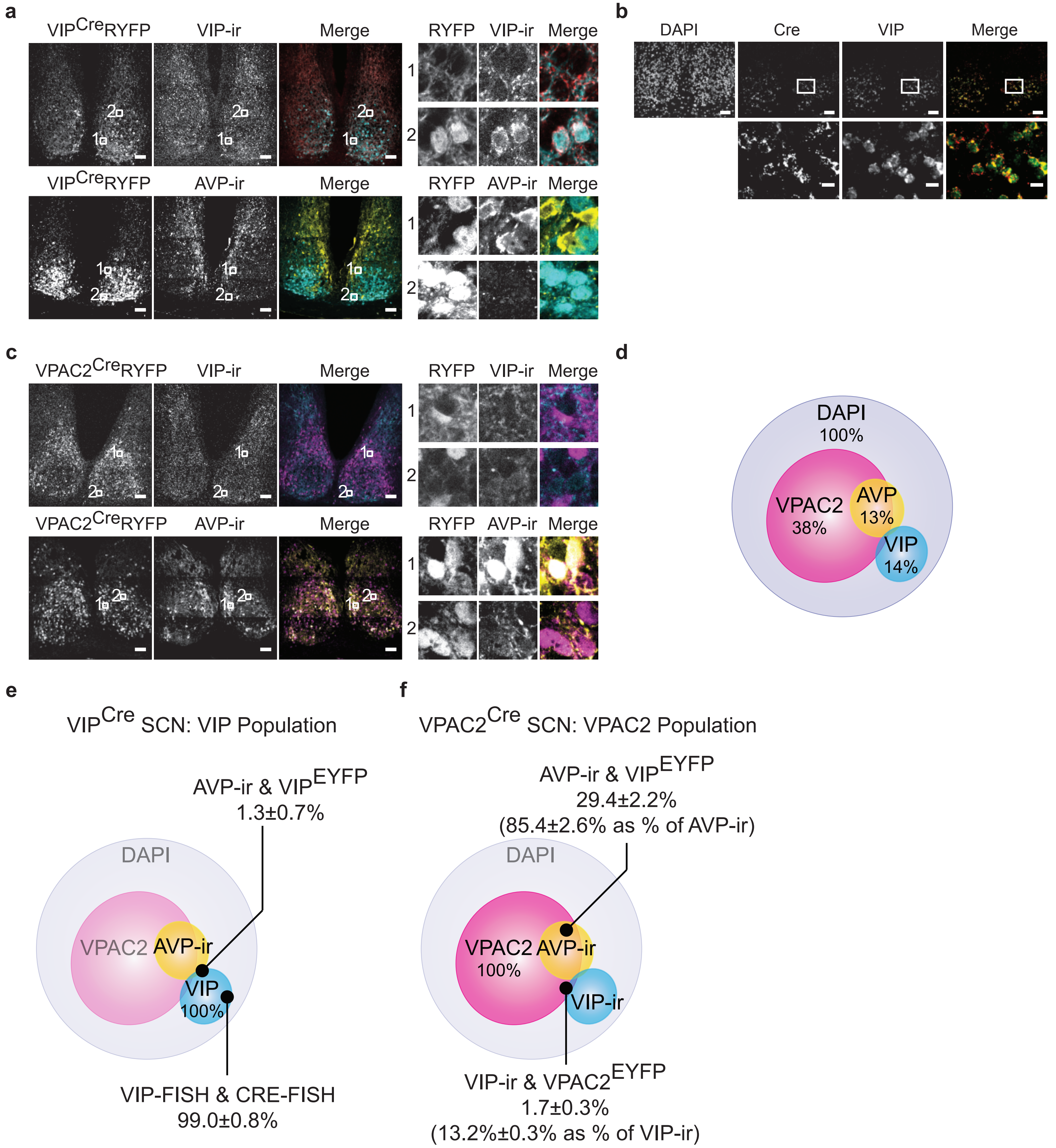

Supplementary Figure 1 | Neurochemical composition of the VIP-VPAC2 neuropeptidergic axis. **(a)** VIP^{Cre}-expressing cells revealed by conditional Rosa-EYFP reporter expression. EYFP-positive cells in the ventral SCN core overlap with VIP-immunoreactivity (-ir) (upper panels), but do not overlap with AVP-ir (lower panels). **(b)** Representative low magnification micrographs of double fluorescent in situ hybridisation (FISH) against VIP and Cre recombinase (upper) showing their complete co-localisation in VIP^{Cre} SCN. Higher magnification views (lower) are taken from the boxed area in the low magnification images. **(c)** VPAC2^{Cre}-expressing cells revealed by conditional Rosa-EYFP reporter expression. EYFP-positive cells in the dorsal SCN shell do not overlap with VIP-ir (upper panels), but do overlap with AVP-ir (lower panels). **(d)** Venn diagram showing the relative proportions of the VIP^{Cre}, VPAC2^{Cre} and AVP-ir cell populations as a percentage of the overall cellular constituents of the SCN as defined by DAPI staining and rounded to the nearest whole number. **(e)** Venn diagram showing the overlap of VIP^{Cre} cell population with VIP-FISH and AVP-ir cell populations expressed as a percentage of VIP^{Cre} cells. **(f)** Venn diagram showing the overlap of VPAC2^{Cre} cell population with VIP-ir and AVP-ir cell populations expressed as a percentage of VPAC2^{Cre} cells. IHC values in **(e)** and **(f)** are mean ±SEM from 3 mice and 2 sections per genotype (VIP-ir and AVP-ir were stained separately in VIP^{Cre} sections and combined in VPAC2^{Cre} sections). FISH values in e are mean ±SEM from 3 mice and 3-4 sections per mouse. Scale bars =50µm (high magnification)/10µm (low magnification).

Supplementary Figure 2

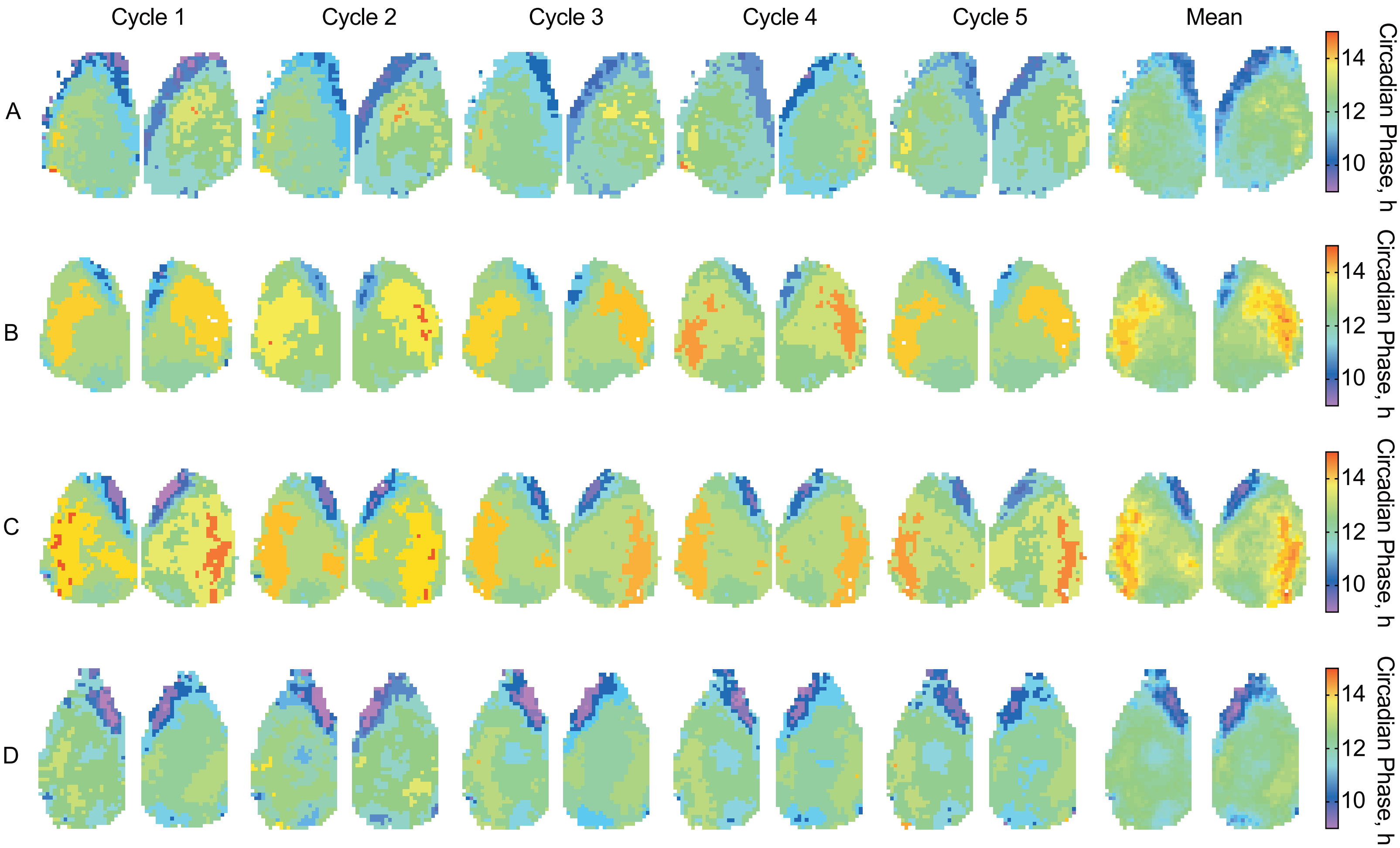

Supplementary Figure 2 | Phase-maps reveal stable SCN spatio-temporal complexity from cycle-to-cycle and slice-to-slice. Four representative SCN slices (A-D) showing the spatiotemporal complexity of the Per2::Luciferase oscillation across 5 cycles as continuous false-coloured phase-maps. Note that the regional phase clusters are present in the same locales across and within the 4 different slices.

Supplementary Figure 3

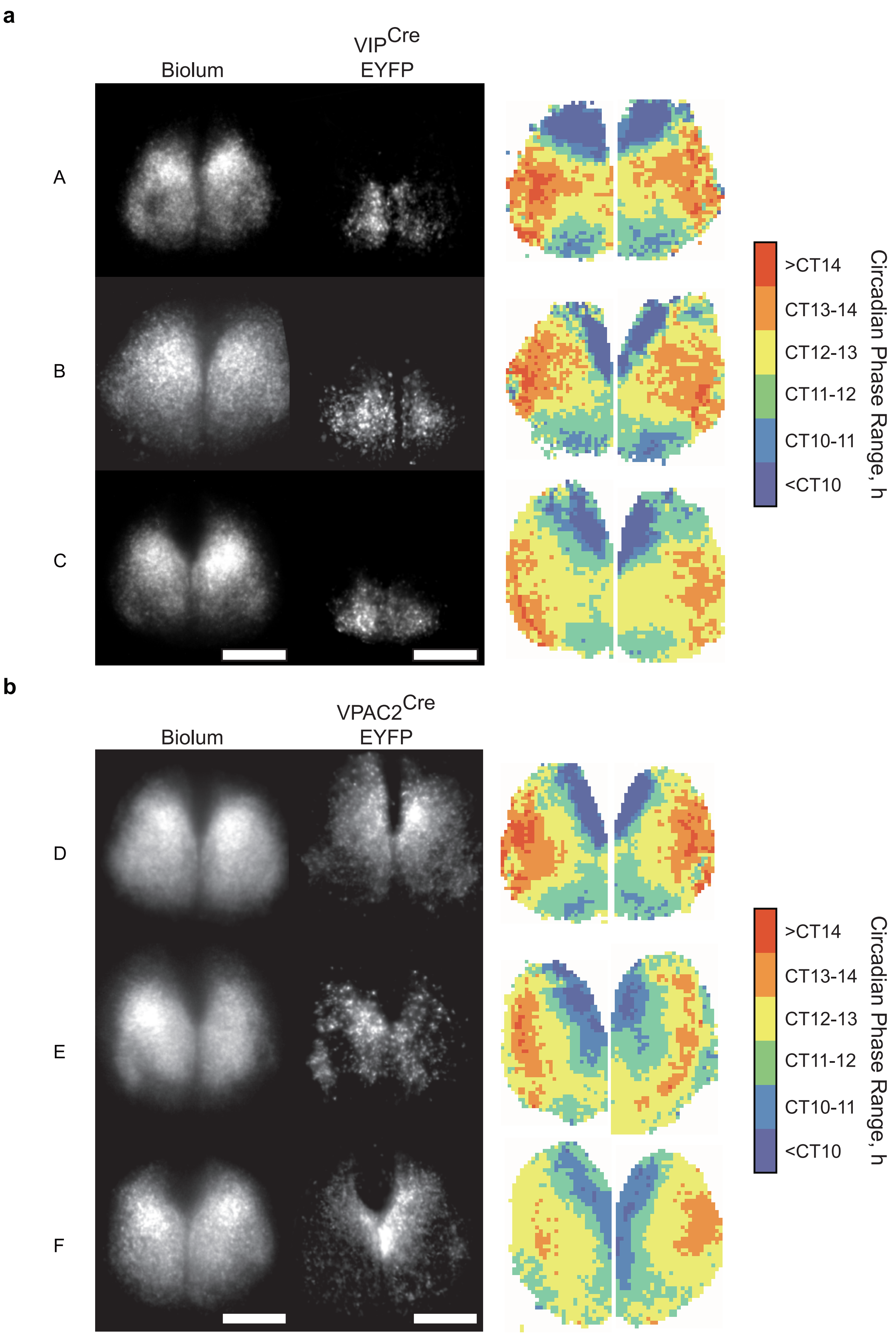

Supplementary Figure 3 | Conditional EYFP expression reveals differential cellular phase territories within SCN PER2::LUC phase-maps. (a) Three representative VIP^{EYFP} SCN slices (A-C) showing bulk bioluminescence (left) alongside VIP-cell territories revealed by conditional expression of EYFP (middle) and Per2::Luciferase categorically-coloured phase-maps (right). Note that the regional VIP^{EYFP} signal aligns with more ventral early phase clusters. The left nucleus of SCN A is reproduced as the VIP^{EYFP} exemplar in Figure 1a. **(b)** Three representative $VPAC2^{EYFP}$ SCN slices (D-F) showing bulk bioluminescence (left) alongside VPAC2-cell territories revealed by conditional expression of EYFP (middle) and Per2::Luciferase categorically-coloured phase-maps (right). Note that the regional $VPAC2^{EYFP}$ signal aligns with more dorsal and lateral late phase clusters. The right nucleus of SCN D is flipped horizontally and reproduced as the $VPAC2^{EYFP}$ exemplar in Figure 1a. Scale bars =250μm.

Supplementary Figure 4

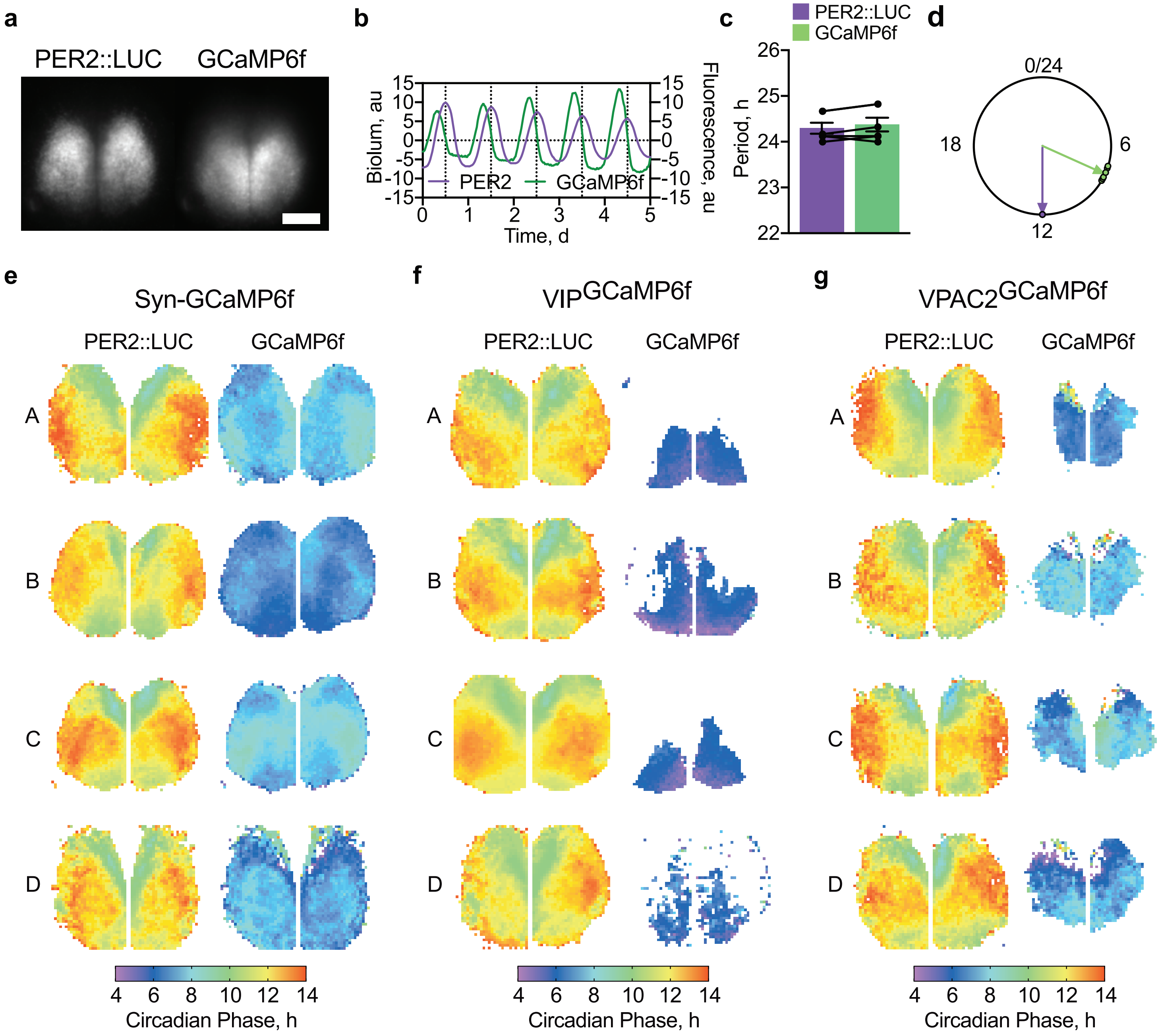

Supplementary Figure 4 | Local spatiotemporal complexity embedded within the pan-neuronal phase wave of the SCN, as revealed by conditional targetting of GCaMP6f.

(a) Representative micrographs of Per2::Luciferase in register with pan-neuronal GCaMP6f. Scale bar =250μm. **(b)** Representative detrended Per2::Luciferase (purple) and pan-neuronal GCaMP6f (green) traces. **(c)** Paired period measures of Per2::Luciferase and pan-neuronal GCaMP6f. **(d)** Rayleigh plot showing circadian phase for pan-neuronal GCaMP6f (green) and Per2::Luciferase (purple, assigned to CT12). **(e-g)** Representative continuous SCN phase-maps from four different SCN per targetting configuration for (left) Per2::Luciferase and (right) neuronal-GCaMP6f restricted pan-neuronally **(e)**, to VIP^{Cre} **(f)** or to $VPAC2^{Cre}$ neurons **(g)**. Maps are aligned in circadian time to the Per2::Luciferase map, centred around CT12. For summary data in **(c)** and **(d)**, n =6 SCN slices. In all plots, bars represent mean ±SEM and individual points represent individual SCN slices. Statistics: **(c)** paired two-tailed t-test. Only significant comparisons ($p<0.05$) are shown. Source data are provided as a source data file.

Supplementary Figure 5

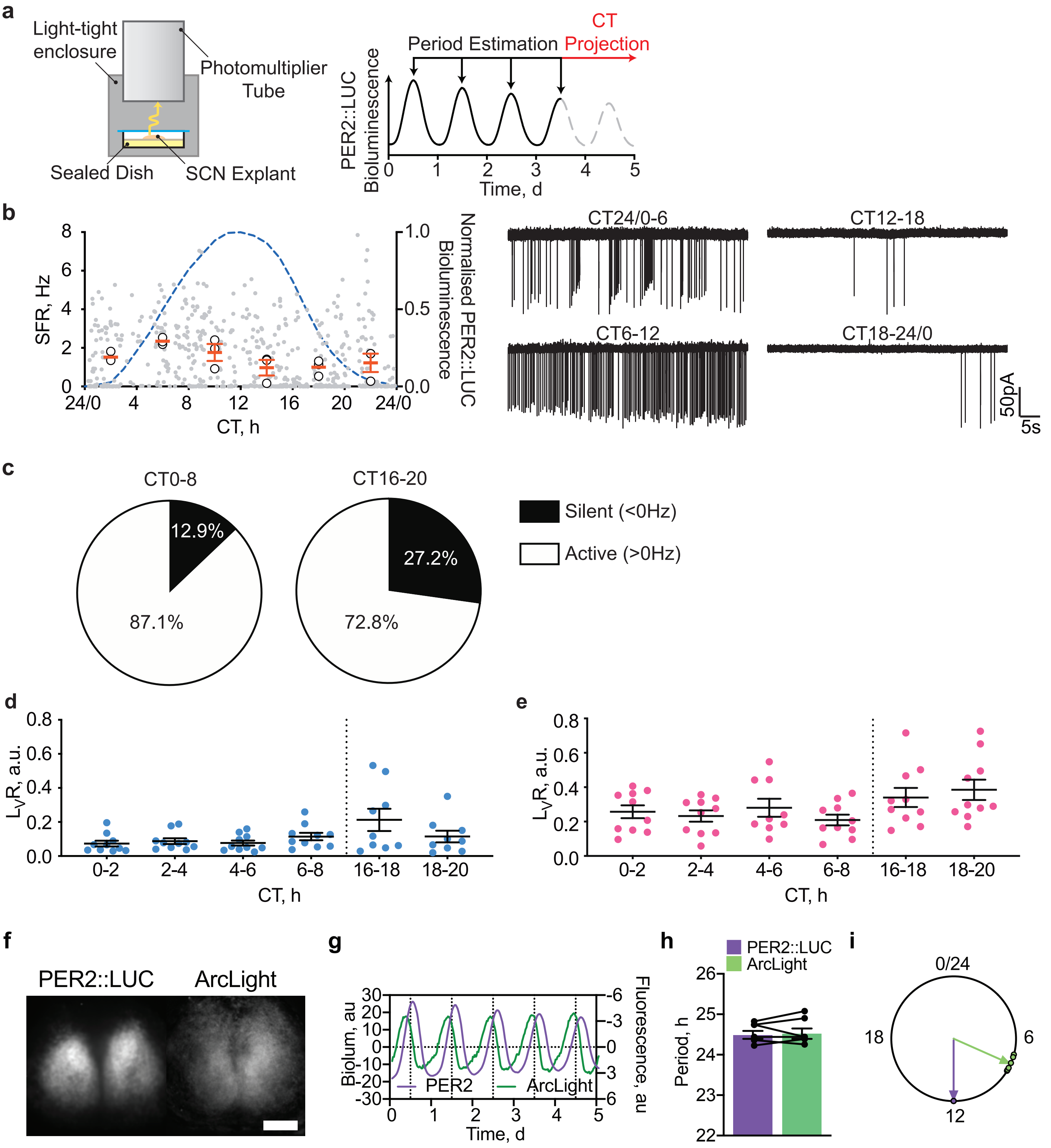

Supplementary Figure 5 | Circadian electrophysiological rhythms in SCN assessed by patch clamp and fluorescent imaging. **(a)** Experimental protocol for the measurement of phase-mapped circadian electrophysiological properties, using a custom-made photomultiplier tube (PMT) apparatus (left) to extrapolate bioluminescent recording times by forward projections of circadian time (right). **(b)** Cell-attached recordings of spontaneous firing rate (SFR) from circadian time-stamped recordings aligned to a representative Per2::Luciferase cycle. Recordings show a circadian oscillation in electrical activity (left) with discharge rates highest during circadian day. Example recordings at different times are shown on the right. Grey points denote discharge rates of individual neurons, hollow circles denote the slice mean for that time bin, red lines represent the mean of the slice means ±SEM, n =8 slices, 605 cells. Welch's ANOVA, time effect p=0.03. **(c)** Pie charts showing the relative proportions of silent (<0Hz) and active (>0Hz) cells during circadian day (CT0-8) and circadian night (CT16-20) from dataset in **(b)**. CT0-8: n =163 cells (4 slices); CT16-20: n =125 cells (3 slices) SCN. **(d and e)** Phase-aligned recordings of L_vR for VIP **(d)** and VPAC2 **(e)** showing time-of-day variations within genotypes. Lines represent mean ±SEM, individual points represent slice mean data. **(f)** Representative micrographs of Per2::Luciferase in register with pan-neuronal ArcLight. **(g)** Detrended Per2::Luciferase (purple) and inverted pan-neuronal ArcLight trace (green). **(h)** Paired period measures of Per2::Luciferase and pan-neuronal ArcLight. Bars represent mean ±SEM, paired points represent individual slices. **(i)** Rayleigh plot of circadian-normalised phase for pan-neuronal ArcLight and Per2::Luciferase (assigned to CT12), points represent individual slices. For **(d-e)**: pooled slice level data for n =10 slices per genotype per time point (except for VIP CT16-18, VIP CT18-20, and VPAC2 CT4-6 where n =9 slices per genotype per time point); for summary data in **(h)** and **(i)**, n = 6 SCN slices. Statistics: **(d and e)** linear mixed model followed by two-way ANOVA with Tukey's correction for multiple comparisons; **(h)** paired two-tailed t-test. Only significant comparisons (p<0.05) are shown. Source data are provided as a source data file.

Supplementary Figure 6

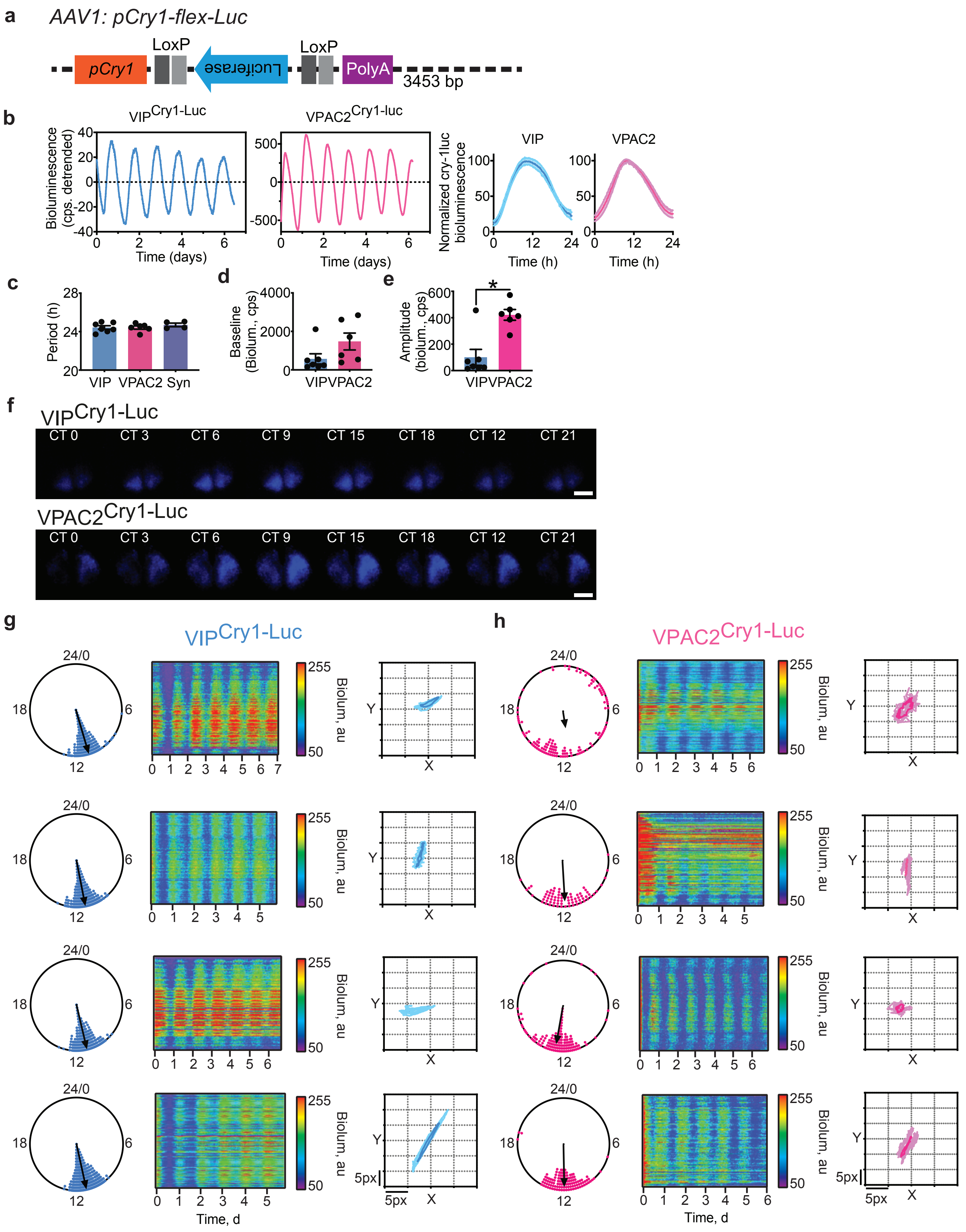

Supplementary Figure 6 | Transcriptional rhythms in VIP- and VPAC2-cells revealed by conditional *pCry1*-Luciferase expression. **(a)** Construct map for the *pCry1*-flex-Luciferase AAV insert. **(b)** Representative detrended PMT traces of conditional *pCry1*-Luc rhythms restricted to VIP- (blue) or VPAC2- (pink) cells (left) alongside mean phase-aligned single cycles (right). **(c)** Summary period data from PMT recordings of VIP^{Cre}, $VPAC2^{Cre}$ or Synapsin-Cre targetted conditional *pCry1*-Luc rhythms. **(d)** Group data for baseline amplitude from PMT data for VIP^{Cre} or $VPAC2^{Cre}$ targetted conditional *pCry1*-Luc rhythms. **(e)** Group data for rhythm amplitude from PMT data for VIP^{Cre} or $VPAC2^{Cre}$ targetted conditional *pCry1*-Luc rhythms (*p=0.014). **(f)** Representative bioluminescent image time-series for VIP^{Cre} (upper) or $VPAC2^{Cre}$ (lower) targetted conditional *pCry1*-Luc signals. Scale bars = 250µm. **(g)** Rayleigh plots (left), Raster plots (middle) and centre of luminescence (CoL) plots for 4 individual VIP^{Cre} targetted conditional *pCry1*-Luc rhythms. **(h)** As in **(g)** but for 4 individual $VPAC2^{Cre}$ targetted conditional *pCry1*-Luc rhythms. Lines and bars are mean ±SEM, represented as error bars or shading. For **(b-e)**: n =7 VIP^{Cre}, n =6 $VPAC2^{Cre}$, n =4 Synapsin-Cre. Statistics: **(c)** One-way ANOVA with Tukey's correction for multiple comparisons; **(d-e)** unpaired two-tailed Mann-Whitney U-test. Only significant comparisons ($p<0.05$) are shown. Source data are provided as a source data file.

Supplementary Figure 7

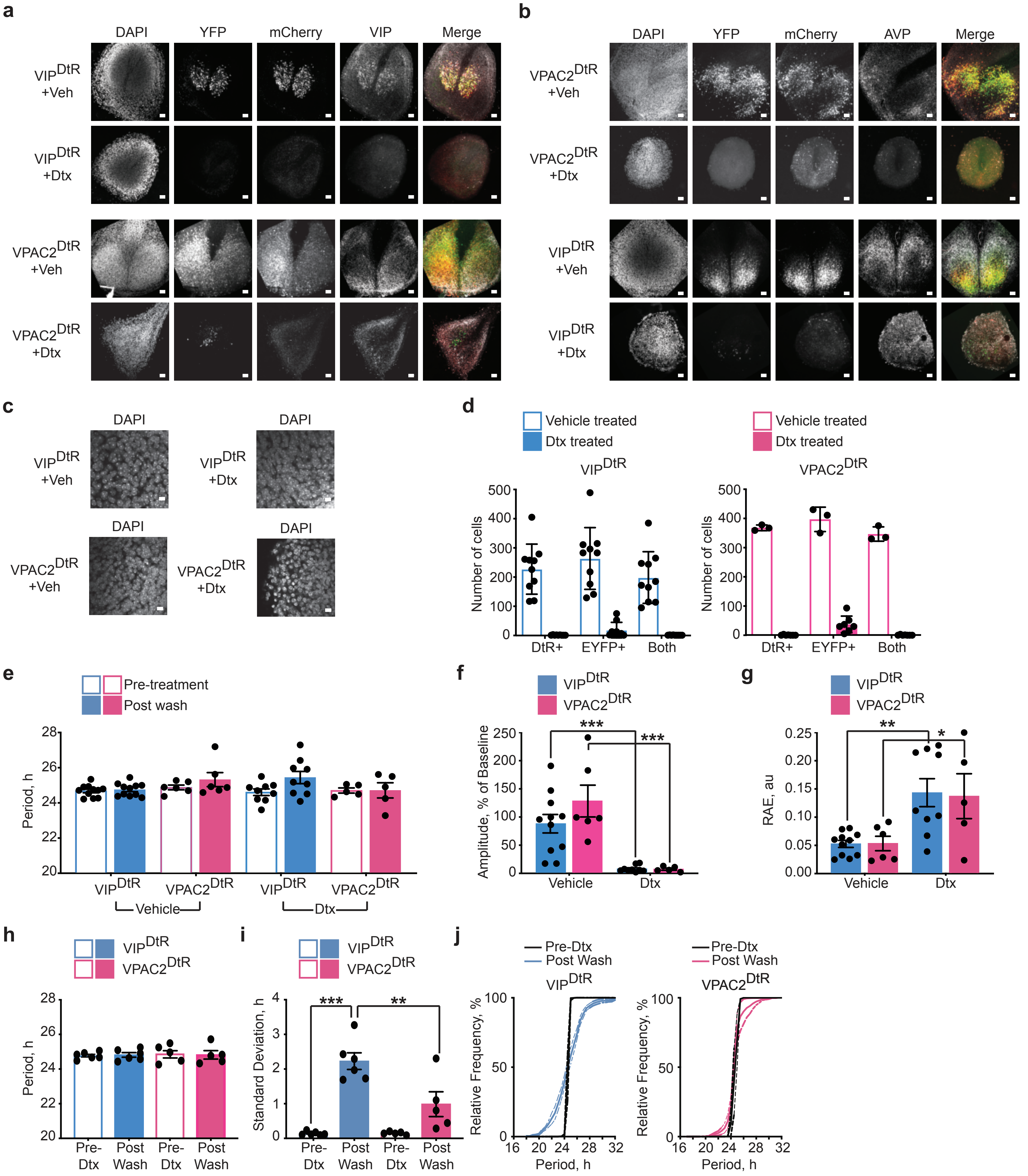

Supplementary Figure 7 | Selective compromise of SCN network time-keeping following specific ablation of VIP^{Cre} or VPAC2^{Cre} cells using Diphtheria toxin. **(a)** SCN slices immunostained for VIP show specific ablation of Diphtheria toxin receptor (DtR)-positive and VIP-immunoreactive (-ir) cells in the presence of diphtheria toxin (Dtx) when DtR expression is restricted to VIP-cells (upper two rows), while VIP^{Cre} ir remains when DtR is targetted to VPAC2^{Cre} cells (lower two rows). **(b)** SCN slices immunostained for AVP show specific ablation of DtR-positive and AVP-ir cells in the presence of Dtx when receptor expression is restricted to VPAC2^{Cre} cells (upper two rows), while AVP-ir remains when DtR is targetted to VIP^{Cre} cells (lower two rows). In **(a and b)**, cell-specific ablation was also confirmed by super-transduction with Cre-conditional EYFP AAVs two weeks post-Dtx (or vehicle). In DtR-expressing, Dtx-treated SCN, EYFP signal was not evident, confirming the absence of Cre-expressing cells. Scale bar =50µm. **(c)** Conditional DtR-expression and Dtx-treatment do not affect nuclear morphology of surviving cells. Scale bar =10µm. **(d)** Cell counts for numbers of DtR-mcherry expressing (DtR), EYFP expressing (EYFP) and DtR-mCherry/EYFP co-expressing (Both) cells for VIP^{Cre} (left, blue) and VPAC2^{Cre} (right, pink) slices following treatment with vehicle (hollow bars) or Dtx (solid bars) treatment. **(e)** Group data for circadian periods of SCN from DtR-mediated conditional ablation experiments showing pre- (hollow bars) and post- (filled bars) treatment periods with DtR restricted to VIP^{Cre} cells (blue) or VPAC2^{Cre} (pink) cells in the presence of vehicle or Dtx. **(f)** Group data for relative amplitude change in PMT recordings induced by treatment of DtR-expressing cells with either vehicle or Dtx (***$p<0.001$). **(g)** Group data for relative amplitude error (RAE) measures from PMT recordings of SCN containing DtR-expressing cells and treated with either vehicle or Dtx (*$p=0.04$, **$p=0.003$). **(h and i)** Group data for bioluminescent imaging of SCN subject to DtR-mediated conditional ablation, showing pre- (hollow bars) and post- (filled bars) treatment mean ROI periods **(h)** and standard deviation of ROI periods **(i)** for DtR restricted to VIP- (blue) or VPAC2- (pink) cells in the presence of Dtx (**$p=0.001$, ***$p=0.0001$). **(j)** Mean cumulative frequency distributions of ROI periods from VIP-ablated (left, blue) and VPAC2-ablated (right, pink) bioluminescent recordings, co-plotted with baseline distributions (black). Lines and bars are mean ±SEM. For **(d)**: n =10 VIP^{Cre}/Veh, n =9 VIP^{Cre}/Dtx, n =3 VPAC2^{Cre}/Veh, n =7 VPAC2^{Cre}/Dtx. For **(e-g)**: n =11 VIP^{Cre}/Veh, n =9 VIP^{Cre}/Dtx, n =6 VPAC2^{Cre}/Veh, n =5, VPAC2^{Cre}/Dtx. For **(h-j)**: n =6 VIP-DtR/Dtx, n =6 VPAC2-DtR-Dtx. Statistics: **(e)** Repeated-measures three-way ANOVA with Sidak's correction for multiple comparisons; **(f-i)** Repeated-measures two-way ANOVA with Sidak's correction for multiple comparisons. Only significant comparisons ($p<0.05$) are shown. Source data are provided as a source data file.

Supplementary Figure 8

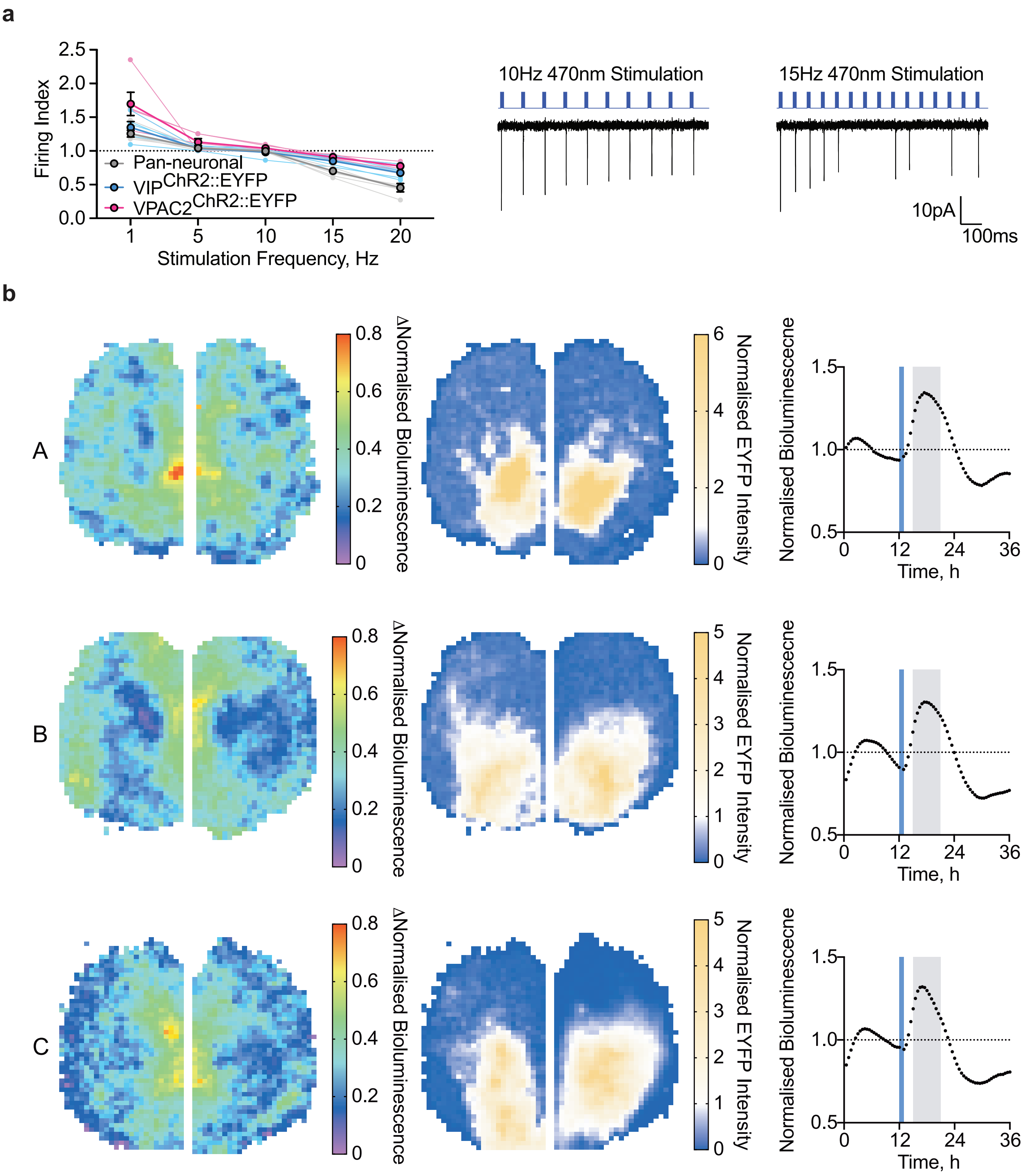

Supplementary Figure 8 | Optogenetic activation controls SCN firing rate, and in VIP^{Cre} cells induces Per2::Luciferase bioluminescence across Cry-null SCN. (a) Calibration curves (left) showing firing index versus stimulation frequency as a measure of reliability of firing induction. In all targetting cases (pan-neuronal (grey), VIP^{Cre} (blue) or VPAC2^{Cre} (pink)), SCN neurons cannot sustain firing rates above 10Hz. Example traces at 10Hz stimulation and 15Hz stimulation are shown on the right of the plot. Individual slice means are shown as lighter circles/lines and the mean of all the slices is shown as darker circles/lines. Circles are mean ±SEM, n =5 for all groups. **(b)** Three independent SCN maps (A-C) showing change in bioluminescence from baseline (left) against EYFP marker for VIP^{Cre} cells (middle) alongside the blue light-induced change in normalised bioluminescence (right). Grey shading indicates the time interval in which post-stimulation bioluminescence change was calculated. Note strong induction of bioluminescence outside the EYFP region. Source data are provided as a source data file.

Supplementary Figure 9

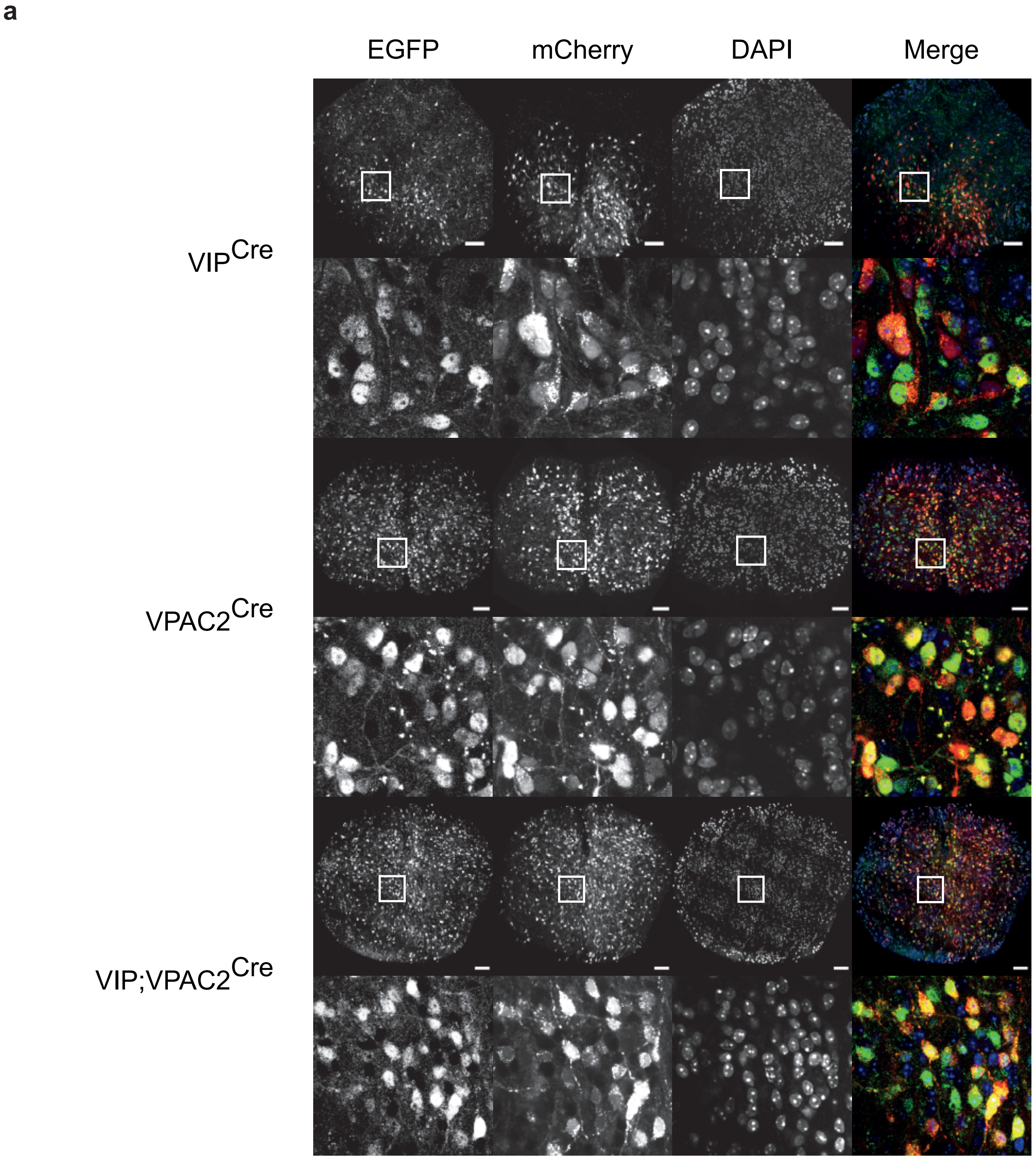

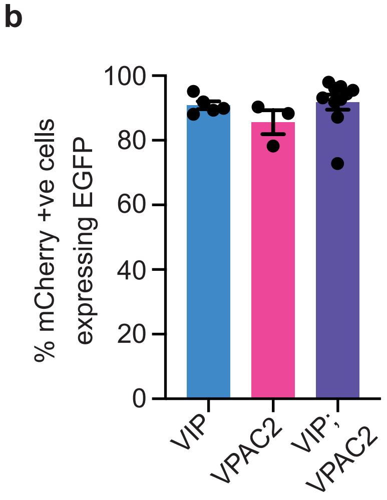

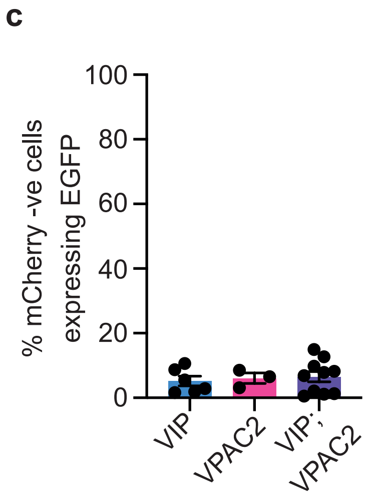

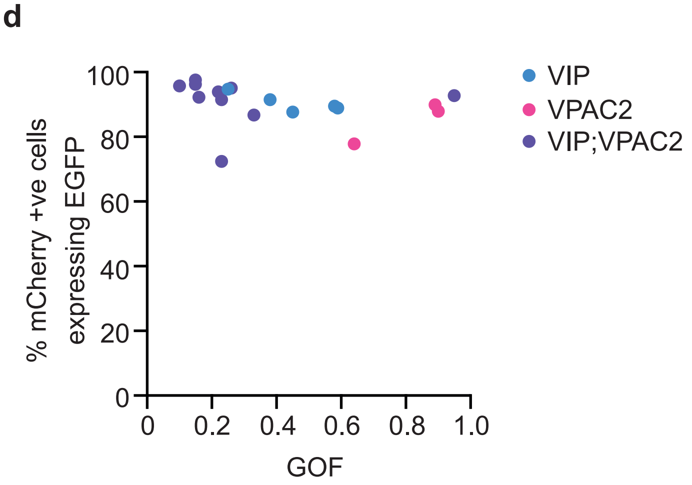

Supplementary Figure 9 | Conditional Cry1::EGFP expression in VIP^{Cre}, VPAC2^{Cre} and VIP^{Cre};VPAC2^{Cre} Cry1-null and Cry1, 2-null SCN. **(a)** Representative false-coloured micrographs of native fluorescence signal from mCherry (red) Cry1::EGFP (green) alongside DAPI (blue), first as individual greyscale channels followed by a false-coloured merge in VIP^{Cre} (top), VPAC2^{Cre} (middle) and VIP^{Cre};VPAC2^{Cre} (bottom) SCN slices following AAV transduction. Scale bar =100μm. Note appropriate spatial distributions of EGFP signal. EGFP+ cells represent (as a proportion of total DAPI staining): VIP^{Cre} 21.3±2.4%, VPAC2^{Cre} 42.0±4.3%, and VIP^{Cre};VPAC2^{Cre} 48.6±3.2%; n =8, 3 and 13 for VIP^{Cre}, VPAC2^{Cre} and VIP^{Cre};VPAC2^{Cre} SCN, as shown in Figure 7d. Close-up images taken from the white boxed areas are shown below the relevant images. Note nuclear localisation of Cry1::EGFP and co-localisation with mCherry, an AAV dependent report of Cre-positive cells. **(b)** Group data showing transduction efficiency of Cry1::EGFP AAV, expressed as the % of Cre-positive cells (revealed by the conditional mCherry report) that are also EGFP-positive. **(c)** Group data for % spill-over of EGFP signal: Proportion of EGFP-positive cells that are not Cre-positive in transduced slices; an inverse measure of conditional mCherry transduction efficiency. **(d)** Scatter plot showing that there is no positive relationship between GOF and transduction efficiency, across all three genotypes. i.e. the strength of circadian rhythm initiation is not related to transduction efficiency. In all plots, bars show mean ±SEM, individual points represent individual SCN slices. For group data: n =5, 3 and 10 for VIP^{Cre} (blue), VPAC2^{Cre} (pink), and VIP^{Cre};VPAC2^{Cre} (purple) SCN slices respectively. Only significant comparisons ($p<0.05$) are shown. Source data are provided as a source data file.

Supplementary Figure 10

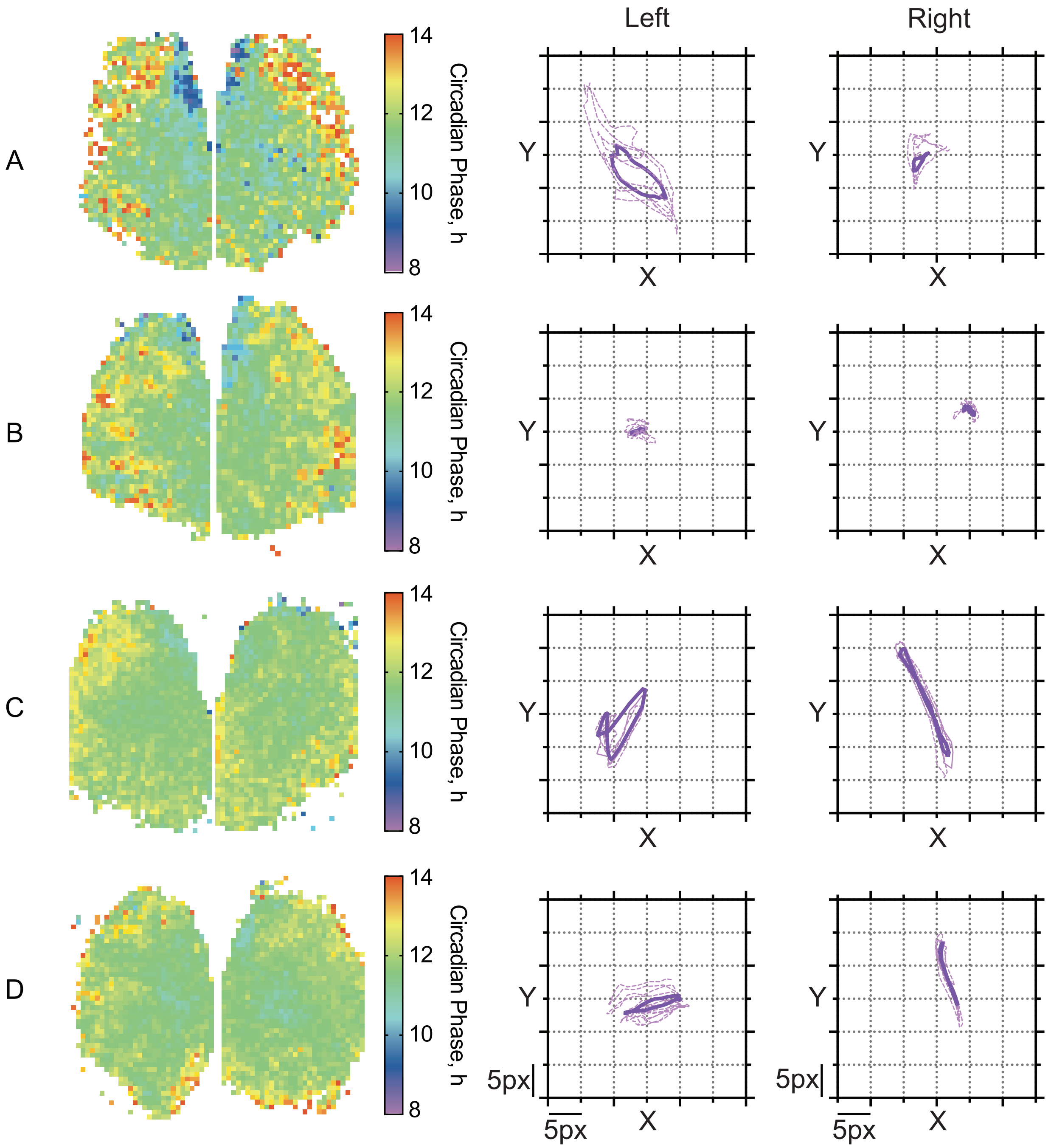

Supplementary Figure 10 | Initiation of rhythmicity in both compartments of the VIP-VPAC2 axis drives circadian spatiotemporal dynamics. False-coloured continuous phase-maps from four independent SCN explants (A-D) (left) alongside their corresponding centre-of-luminescence (CoL) plots (right) showing the initiation of spatiotemporal dynamics to Per2::Luciferase expression following simultaneous Cry1::EGFP expression in VIP^{Cre} and $VPAC2^{Cre}$ cells. The corresponding CoL plots (right) show individual cycles (dashed purple) overlaid with the mean of the individual cycles (solid purple) from left and right SCN.

Supplementary Figure 11

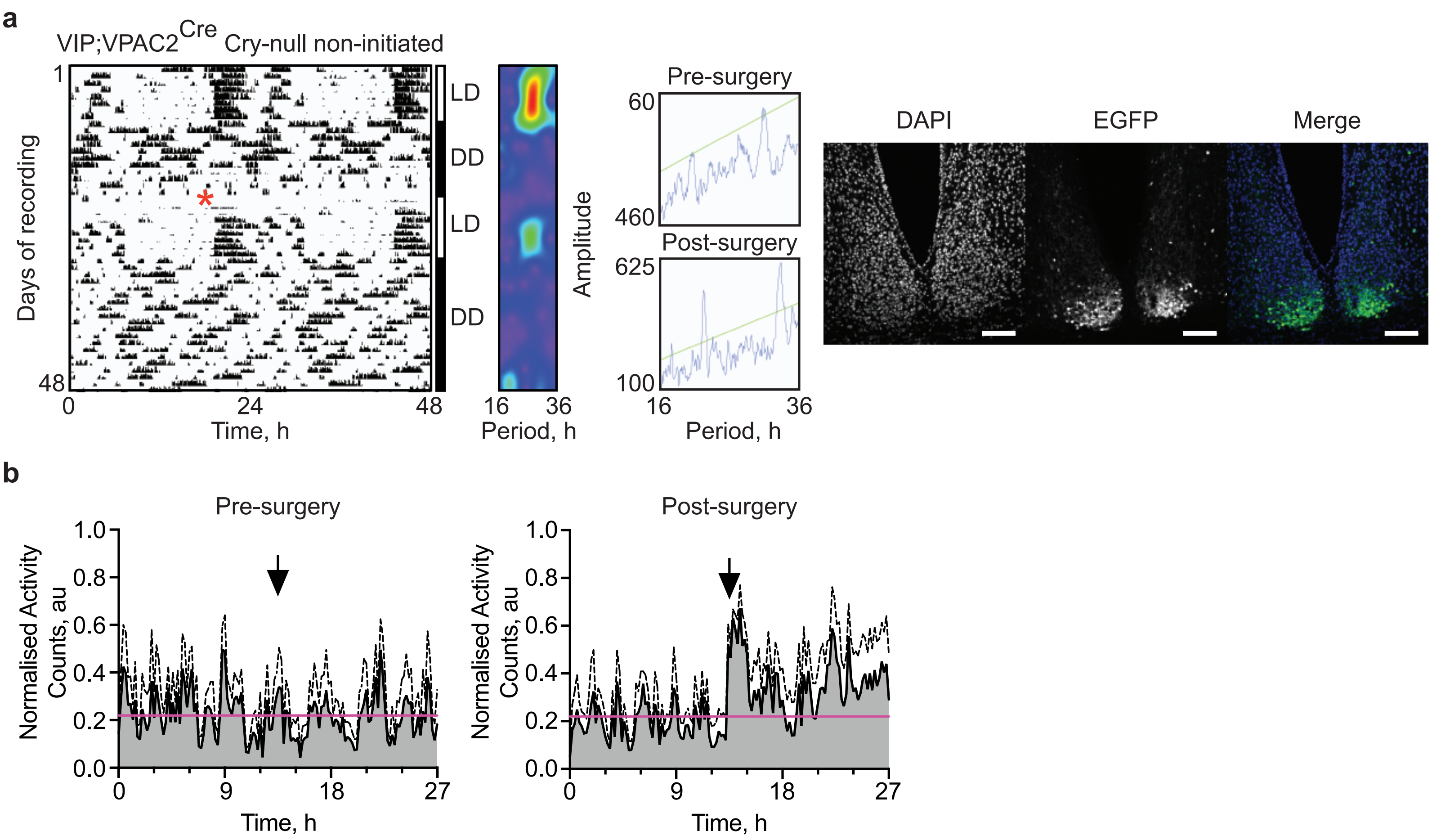

Supplementary Figure 11 | Initiation of rhythmicity in both the VIP-VPAC2 cells of the SCN *in vivo* initiates behavioural rhythmicity. **(a)** Representative double-plotted wheel-running traces (plotted on a 24h time base) showing failed initiation of rhythmic behaviour in a VIP;VPAC2^{Cre} Cry-null mouse. Before surgery (denoted by red asterisks), the behaviour is arrhythmic and this arrhythmic behaviour persists post-surgery as quantified in the wavelet and amplitude plots to the right of the actogram. Post-hoc confocal imaging (right) reveals that this mouse was mis-targetted with the bulk of Cry1::EGFP expression restricted to the ventral core region of the SCN. Scale bars =100µm. **(b)** Average normalised activity profiles for the last 7 days of recording pre- (left) and post-surgery (right) shown as mean ±SEM. The activity of each mouse is aligned at activity onset (indicated by the black arrow), and the mean activity from the pre-surgery condition (subjected to the same relative alignment) is plotted as a horizontal line (pink) (n = 5 mice). Source data are provided as a source data file.

Supplementary Figure 12

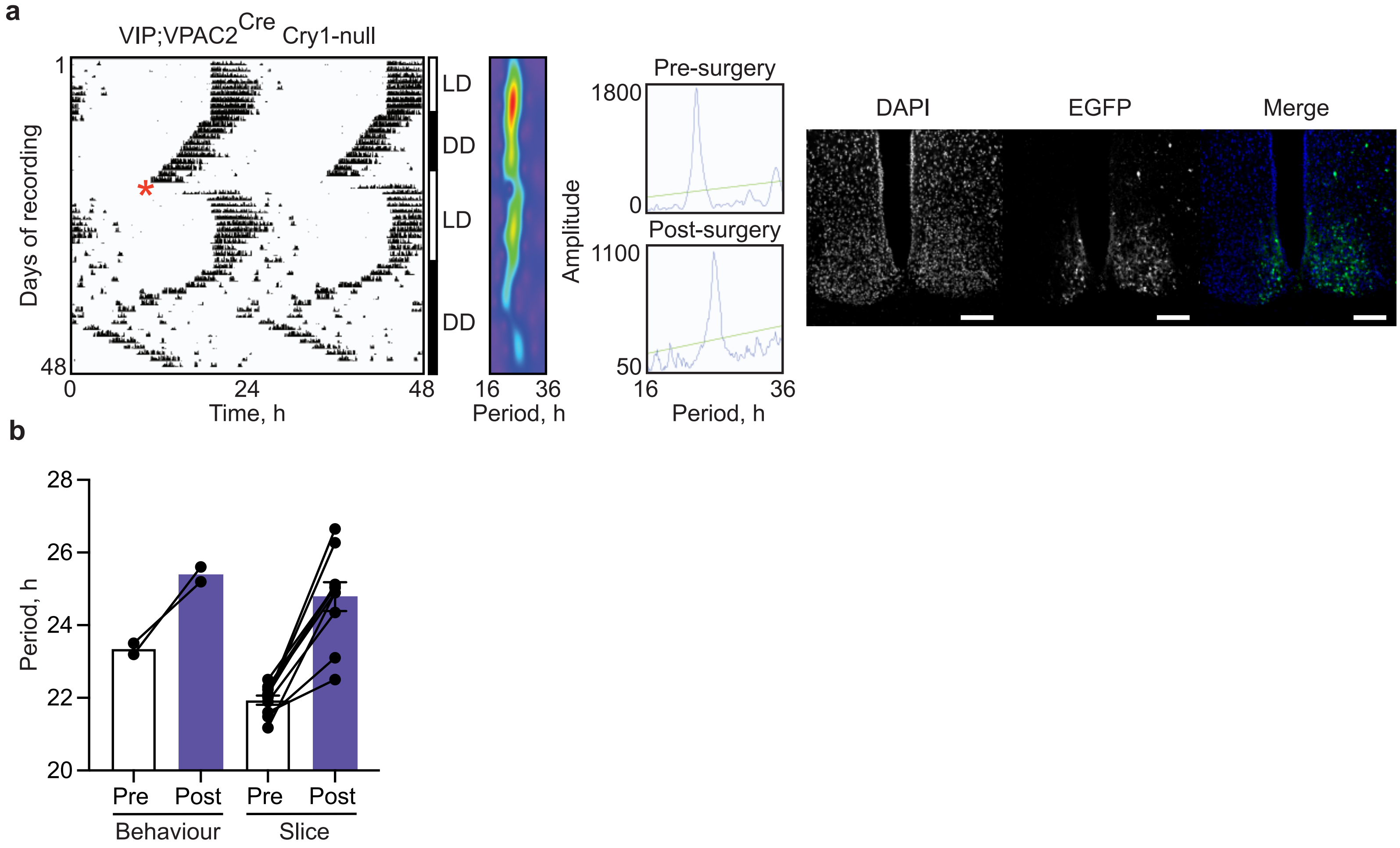

Supplementary Figure 12 | Conditional Cry1 expression in the VIP-VPAC2 cellular axis is sufficient to determine the period of circadian behaviour. (a) Representative double-plotted wheel-running traces (plotted on a 24h time base) showing successful initiation of rhythmic behaviour in a VIP;VPAC2^{Cre} Cry1-null, Cry2-competent mouse. Before surgery (denoted by red asterisks), the behaviour is rhythmic with a short period while following surgery, the behavioural period in this mouse lengthened as shown by the wavelet and amplitude plots to the right of the actogram. Post-hoc confocal imaging (right) reveals that this mouse was successfully targetted with the Cry1::EGFP expression spanning the ventral core and dorsal shell regions of the SCN. Scale bars =100µm. **(b)** Behavioural periods of VIP;VPAC2^{Cre} Cry1-null; Cry2-competent mice pre- and post-surgery (left) plotted alongside *ex vivo* SCN slice data pre- and post-AAV administration (right, reproduced from Figure 6d) (n =2 mice/10 SCN). Paired individual points represent individual mice/SCN slices; bars represent mean ± SEM. Only significant comparisons ($p<0.05$) are shown. Source data are provided as a source data file.

Supplementary Table 1 | Numbers of individual SCN subjected to each treatment at each phase in the optogenetic phase response curves (PRCs), related to Figure 5c,e and f.

Circadian Phase	$VIP^{ChR2::EYFP}$		$VPAC2^{ChR2::EYFP}$	
	470nm	625nm	470nm	625nm
CT0-3	6	8	8	6
CT3-6	6	5	5	7
CT6-9	6	8	8	5
CT9-12	6	5	5	7
CT12-15	11	7	9	6
CT15-18	6	6	7	6
CT18-21	7	8	6	6
CT21-24	8	6	8	6
