# Supplementary material for: The VIP-VPAC2 neuropeptidergic axis is a cellular pacemaking hub of the suprachiasmatic nucleus circadian circuit
Source: Nat Commun. 2020 Jul 7;11:3394. doi: 10.1038/s41467-020-17110-x (PMC7341843; doi:10.1038/s41467-020-17110-x)
Supplement: Supplementary file 3 — Reporting Summary [file 41467_2020_17110_MOESM3_ESM.pdf]

nature research

Corresponding author(s): Michael Hastings

Last updated by author(s): May 26, 2020

Reporting Summary

Nature Research wishes to improve the reproducibility of the work that we publish. This form provides structure for consistency and transparency in reporting. For further information on Nature Research policies, see our Editorial Policies and the Editorial Policy Checklist.

Statistics

For all statistical analyses, confirm that the following items are present in the figure legend, table legend, main text, or Methods section.

n/a	Confirmed	
☐	☒	The exact sample size (*n*) for each experimental group/condition, given as a discrete number and unit of measurement
☐	☒	A statement on whether measurements were taken from distinct samples or whether the same sample was measured repeatedly
☐	☒	The statistical test(s) used AND whether they are one- or two-sided<br>*Only common tests should be described solely by name; describe more complex techniques in the Methods section.*
☒	☐	A description of all covariates tested
☐	☒	A description of any assumptions or corrections, such as tests of normality and adjustment for multiple comparisons
☐	☒	A full description of the statistical parameters including central tendency (e.g. means) or other basic estimates (e.g. regression coefficient) AND variation (e.g. standard deviation) or associated estimates of uncertainty (e.g. confidence intervals)
☐	☒	For null hypothesis testing, the test statistic (e.g. *F*, *t*, *r*) with confidence intervals, effect sizes, degrees of freedom and *P* value noted<br>*Give P values as exact values whenever suitable.*
☒	☐	For Bayesian analysis, information on the choice of priors and Markov chain Monte Carlo settings
☒	☐	For hierarchical and complex designs, identification of the appropriate level for tests and full reporting of outcomes
☒	☐	Estimates of effect sizes (e.g. Cohen's *d*, Pearson's *r*), indicating how they were calculated

Our web collection on statistics for biologists contains articles on many of the points above.

Software and code

Policy information about availability of computer code

Data collection	AxographX, Olympus cellM, Xcellence rt and cellSens, Micromanager (ImageJ), ClockLab
Data analysis	Microsoft Excel, AxographX, Igor Pro, FIJI (ImageJ), RStudio running R (version 3.6.1), Graphpad Prism 8, ClockLab, BioDARE

For manuscripts utilizing custom algorithms or software that are central to the research but not yet described in published literature, software must be made available to editors and reviewers. We strongly encourage code deposition in a community repository (e.g. GitHub). See the Nature Research guidelines for submitting code & software for further information.

Data

Policy information about availability of data

All manuscripts must include a data availability statement. This statement should provide the following information, where applicable:

- Accession codes, unique identifiers, or web links for publicly available datasets
- A list of figures that have associated raw data
- A description of any restrictions on data availability

The source data underlying Figures 1d and f, 2b-g, j-k, 3e, g and i, 4g, 5c, e-h, k-l, 6a-d, 7b-f, and 8c-f and the source data underlying Supplementary Figures 4c-d, 5b, d-e, h-l, 6c-e, 7d-j, 8a, 9b-d, 11b and 12b are provided as a Source Data file. Other data and biological materials are available from the corresponding author upon reasonable request.

Field-specific reporting

Please select the one below that is the best fit for your research. If you are not sure, read the appropriate sections before making your selection.

- [x] Life sciences
- [ ] Behavioural & social sciences
- [ ] Ecological, evolutionary & environmental sciences

For a reference copy of the document with all sections, see nature.com/documents/nr-reporting-summary-flat.pdf

Life sciences study design

All studies must disclose on these points even when the disclosure is negative.

Sample size

Sample sizes, statistical power and variance are routinely determined in G*Power and Prism 8.

Data exclusions

In behavioural experiments, mice were excluded from statistical analysis if surgical targeting of the SCN was shown to have been inefficient (missed) based on post-hoc histology, as described in the materials and methods. An example is nevertheless provided in the supplementary information.
In electrophysiological experiments, cells were excluded if the series resistance between the beginning and end of the recordings changed by more than 25%, as described in the materials and methods.
In both cases, exclusion criteria were pre-determined.

Replication

Replication was conducted on independent batches of brain tissue slices obtained from genetically independent mouse litters. Behavioural experiments were conducted on individual mice from two different cohorts. Across the whole study, at least three replicates were performed (except in the case of the supplemental behavioural Cry-complementation (Supplementary Figure 12) where n=2, no further statistical analysis was performed on this cohort).

Randomization

Once genotypes were determined, brain tissue slices were assigned at random to treatments.

Blinding

It was not always possible to blind experiments as genotypes of tissue slices and mice, the AAV treatments and other treatments had to be known to the investigators to ensure correct experimental design.

Behavioural data was assessed blinded: two investigators blind to genotype and targeting of mice independently assessed activity patterns for rhythm initiation or period change. The targeting, genotypes and analysis results were then unblinded to both investigators. There was full agreement between the assessments of the two investigators.

Reporting for specific materials, systems and methods

We require information from authors about some types of materials, experimental systems and methods used in many studies. Here, indicate whether each material, system or method listed is relevant to your study. If you are not sure if a list item applies to your research, read the appropriate section before selecting a response.

Materials & experimental systems

n/a	Involved in the study
[ ]	[x] Antibodies
[x]	[ ] Eukaryotic cell lines
[x]	[ ] Palaeontology and archaeology
[ ]	[x] Animals and other organisms
[x]	[ ] Human research participants
[x]	[ ] Clinical data
[x]	[ ] Dual use research of concern

Methods

n/a	Involved in the study
[x]	[ ] ChIP-seq
[x]	[ ] Flow cytometry
[x]	[ ] MRI-based neuroimaging

Antibodies

Antibodies used

Rabbit anti-AVP: Peninsula T-4563 (1:1000)
Rabbit anti-VIP: Immunostar 20077 (1:750)
Guinea pig anti-VIP: Peninsula T-5030 (1:1000)
Mouse monoclonal anti-DIG-POD: Abcam, ab420 (1:100)
Mouse monoclonal anti-FLU-POD: Abcam, ab6213 (1:100)

Validation

Validation was not carried out in-house as the three antisera and two antibodies we used are well characterised in the literature (see references on manufacturer's websites). Moreover, they behaved as expected by marking the appropriate genetically tagged (by recombinase expression) cells ands regions and thus delineating the core and shell of the SCN consistent with other studies.

Animals and other organisms

Policy information about studies involving animals; ARRIVE guidelines recommended for reporting animal research

Laboratory animals	All mice used in the study were routinely backcrossed onto a C57Bl6 background.<br>Mice had the following genotypes either alone or in combination: PER2::LUC, VIP-Cre, VPAC2-Cre, Cry1-null, and Cry1,2-null (Cry-null). Mice carrying these alleles were bred in-house as described in the text.<br><br>Brain tissue slices were derived from mouse pups of both sexes, ca. 10 days of age.<br><br>Adult mice aged between 10-14 weeks with the genotypes VIP-Cre/VPAC2-Cre/Cry-null and VIP-Cre/VPAC2-Cre/Cry1-null were obtained under the same breeding strategy as pups.
Wild animals	No wild animals were used in the study.
Field-collected samples	No field-collected samples were used in the study.
Ethics oversight	UK Home Office Project and Personal Licences, protocols approved by Local Ethics Board (LMB AWERB) overseen by designated animal welfare officers (NACWOs).

Note that full information on the approval of the study protocol must also be provided in the manuscript.
