# Supplementary material for: The VIP-VPAC2 neuropeptidergic axis is a cellular pacemaking hub of the suprachiasmatic nucleus circadian circuit
Source: Nat Commun. 2020 Jul 7;11:3394. doi: 10.1038/s41467-020-17110-x (PMC7341843; doi:10.1038/s41467-020-17110-x)
Supplement: Supplementary file 4 — Description of Additional Supplementary Files [file 41467_2020_17110_MOESM4_ESM.pdf]

Description of Additional Supplementary Files

File Name: Supplementary Movie 1

Description: CCD time-lapse video of Per2::Luciferase bioluminescent emissions from an explant SCN culture recorded over 5-6 days. Note the stereotypical spatiotemporal waves of Per2::Luciferase bioluminescence that persist from cycle-to-cycle.
